# Age-related obesity and inflammaging in cats

**DOI:** 10.3389/fvets.2025.1639055

**Published:** 2025-10-13

**Authors:** Miki Kobayashi, Motoo Kobayashi

**Affiliations:** Seijo Kobayashi Veterinary Clinic, Tokyo, Japan

**Keywords:** aging, inflammaging, cats, obesity, senescence-associated secretory phenotype

## Abstract

Aging is characterized by chronic systemic inflammation accompanied by cellular senescence, immunosenescence, organ dysfunction, and age-related diseases. A chronic low-grade pro-inflammatory state known as “inflammaging” accelerates age-related diseases such as obesity, diabetes, vascular diseases, and certain types of cancer. Senescent cells drive age-related tissue dysfunction partially by inducing a chronic senescence-associated secretory phenotype (SASP) associated with various diseases. Obesity and insulin resistance change with advancing age and are linked to low-grade chronic inflammation, leading to age-related diseases. Obesity results in significant changes in the adipokine profile, such as reduced levels of anti-inflammatory adipokines, e.g., adiponectin. Cats are more prone to obesity than dogs owing to the unique characteristics of their glucose and lipid metabolism. Severely obese cats show excessive visceral fat accumulation, significantly increased triglyceride, free fatty acids, and TNF-α plasma concentrations as pro-inflammatory markers, and a significant decrease in adiponectin. Aged obese cats with excessive visceral fat exhibit fatty liver and enlarged adipocytes with macrophage infiltration. A healthy lifestyle is recognized as the most effective way to maintain health and fight aging. Aging is inevitable in animals; however, delaying the onset of age-related disease through adequate interventions at the early stages of SASP induction is possible. Adequate nutrition, moderate exercise, and a good mental state can effectively prevent age-related obesity in cats.

## 1 Introduction

As aging progresses, the ability of the animal body to resolve inflammation is reduced significantly, resulting in an imbalance between proinflammation and anti-inflammation. This results in a chronic low-grade pro-inflammatory state known as “inflammaging,” which accelerates age-related diseases like obesity, diabetes, vascular diseases, and certain types of cancer (1). Inflammaging occurs in senescent tissues and is involved in the development of age-related diseases (2). Oxidative stress (OS) is associated with various age-related conditions, including sarcopenia and frailty (3), and OS-induced aging and associated disorders cause soft tissue deterioration and homeostatic imbalances (4, 5). Moreover, stressed senescent cells exhibit an altered secretome, referred to as the senescence-associated secretory phenotype (SASP), which results in the secretion of pro-inflammatory cytokines (6).

The increase in the prevalence of overweight and obesity represents a worldwide phenomenon that is associated with various chronic diseases such as type-2 diabetes (T2D), cancer, rheumatoid arthritis and osteoarthritis (OA), cognitive impairment and dementia, and those affecting the cardiovascular (CV) system (7). Obesity superimposed on aging (age-related obesity) represents an additional risk factor for the older age group in which the prevalence of chronic diseases, as well as the occurrence of complications, increases (8, 9). In cats, whose glucose and lipid metabolism differ from that in dogs, obesity and its associated diseases increase significantly with age (10, 11). In this review, we outline chronic inflammation as the basic pathophysiology of age-related diseases and discuss the relationship between age-related obesity and inflammaging in cats.

## 2 Senescence-associated secretory phenotype and inflammaging

Aging is characterized by chronic systemic inflammation accompanied by cellular senescence, immunosenescence, organ dysfunction, and age-related diseases (12). Cellular senescence is a state of permanent cell proliferation arrest induced by persistent DNA damage and other stress-induced signals. Cellular senescence has since been reported not only in cultured cells but also *in vivo* in cells of various organisms, ranging from yeast to mammals (13). *In vivo*, cellular senescence is induced by DNA damage-associated stress. The role and mechanism underlying senescence-associated secretory phenotypes (SASP) have been increasingly recognized, as they are suspected to be associated with various diseases (14). Many senescent cells secrete a wide spectrum of bioactive factors, including inflammatory cytokines, chemokines, growth factors, matrix metalloproteases, lipids, nucleotides, extracellular vesicles, and soluble factors, termed SASP (15). The combination of these molecules forms the SASP, which determines various processes in the body associated with regeneration (16), tissue remodeling (17), inflammation (14), and carcinogenesis (18). The SASP is a dynamic process that can be divided into several phases. The first phase starts immediately after DNA damage, followed by an early SASP phase characterized by increased synthesis spanning several days. Within 4–10 days, the secretion of most SASP factors increases through autocrine exposure, leading up to the mature phase of the SASP (19). SASP regulation occurs at both the transcriptional and post-transcriptional levels. Nuclear factor kappa B (NF-κB) plays a key role in regulating the expression of genes that are the main components of the SASP (20). Senescent cell accumulation and long-term SASP secretion may result in disrupted tissue function, accelerated aging, and the development of age-related pathologies (21). Mitochondrial dysfunction is an often-unappreciated hallmark of cellular senescence which plays important roles not only in the senescence growth arrest but also in the development of the SASP and resistance to cell-death (22).

The term “inflammaging,” first used by Franceschi et al. in 2000, is associated with chronic sub-clinical inflammatory processes and biological aging (23). The SASP phenotype has been proposed as the underlying cause of inflammaging and comprises various soluble factors, including pro-inflammatory mediators (e.g., IL-6 and IL-8) and matrix-degrading molecules characterized by the release of pro-inflammatory cytokines (24). Senescent cells exhibit molecular (e.g., senescence marker expression) and morphological features (e.g., enlarged or flattened cells) (25).

## 3 Inflammaging and age-related diseases

Aging is the strongest risk factor for most chronic diseases, including obesity. Central obesity and inflammation have consistently been found to be strongly associated with the severity and future risk of severe multimorbidity. The pro-inflammatory state of aging has been suggested to be a proxy biomarker of the pace of aging. Strong epidemiological evidence suggests that elevated levels of pro-inflammatory markers in older animals are associated with the risk of developing most diseases typical of aging (26). The systemic consequences of aging on the development of aging phenotypes can be roughly summarized into four major domains: (1) changes in body composition, (2) an imbalance between energy availability and demand, (3) dysregulation of signaling networks that maintain homeostasis, and (4) neurodegeneration with impaired neuroplasticity (26). Age-related changes in body composition and physical fitness are among the most apparent and unavoidable effects of aging, and cause metabolic dysfunction (Table 1). Visceral fat, which is responsible for many obesity-related pathologies and an independent risk factor for coronary artery disease, stroke, and death, continuously accumulates and is reflected in an increase in waist circumference throughout life (27). Evidently, all organs experience some changes in tissue composition throughout life, and the related changes are directly associated with sub-clinical and clinical pathology, including neurodegeneration (28), physical frailty, increase in fibro-connective build-up in muscles, and demineralization and loss of bone strength (29).

**Table 1:** Age-related changes contributing metabolic dysfunction.

Physical frailty
Physical inactivation
Ectopic lipid deposition
Visceral adiposity
Insulin resistance
Senescence-associated secretory phenomenon (SASP)

The balance between energy availability and demand is tightly regulated, and ATP is constantly resynthesized because its storage is sufficient for only a few seconds (30). In muscle cells and neurons, this stability is co-adjuvanted by the phosphocreatine buffering system, which accumulates chemical energy to be promptly used when demand suddenly increases. Most energy muscles use is generated through aerobic metabolism; hence, energy consumption can be estimated indirectly from oxygen consumption. Older individuals with multiple comorbidities have less available energy and require more energy at rest and during physical activity. The amount of energy used at rest decreases with age largely because of a loss of lean body mass but declines less in those with multiple chronic conditions (physical inactivation) (31). Hence, sick older individuals use most of their available energy to perform activities essential to daily living.

A mild pro-inflammatory state develops in most aging individuals, reflected by high levels of pro-inflammatory markers, such as IL-6 and C-reactive protein (CRP) (32). These hormones, inflammatory biomarkers, and antioxidants are part of complex signaling networks that control homeostasis, and individual biomarker levels may reflect adaptations within homeostatic feedback loops rather than causative factors. The number of neurons also declines throughout life, as neurons generally stop reproducing shortly after birth (31). With aging, microglia acquire a predisposition to reactive inflammation, and brain tissue from older individuals exhibits higher levels of pro-inflammatory cytokines and lower levels of anti-inflammatory cytokines than those in the brain tissue of younger individuals. Higher inflammation has been associated with lower cognition and reduced neuronal plasticity, which are expressed as reduced capacities for adaptation and compensation (33). Clinically, inflammaging is characterized by increased blood levels of several inflammatory biomarkers, including CRP, IL-6, IL-8, and TNF-α (34). Furthermore, serum IL-6 levels also predict incident disability and frailty (35).

Obesity and insulin resistance are altered with advancing age and are linked to low-grade chronic inflammation, leading to age-related systemic metabolic dysfunction, physical limitation, and frailty (36). Mitochondrial hormesis may also play a role in aging, and mild mitochondrial toxicity may trigger beneficial compensatory responses that improve cellular fitness (37). Resveratrol and metformin, which inhibit cellular energy metabolism by increasing AMP levels, activating AMP-activated protein kinase (AMPK), and decreasing oxygen uptake, are possible examples of this (38). Obesity has become a prominent health problem globally and is closely associated with many chronic diseases, such as diabetes mellitus, cardiovascular diseases, and certain types of cancer (39). Obesity develops when energy intake exceeds energy expenditure and is characterized by excessive adipose tissue (AT) accumulation. When AT reaches its maximum capacity for energy storage, it releases free fatty acids (FFA), causing ectopic lipid deposition in other tissues, such as the liver, skeletal muscle, and vasculature. Adipose tissue shows increased macrophage infiltration during the development of obesity (40, 41). Consequently, these AT macrophages secrete high levels of pro-inflammatory cytokines, resulting in obesity-associated chronic low-grade inflammation and impaired insulin signaling (42).

Obesity results in significant changes in the adipokine profile, creating a shift toward elevated levels of pro-inflammatory adipokines, such as leptin and resistin, and reduced levels of anti-inflammatory adipokines, such as adiponectin (43). Obesity is also associated with increased perivascular fat, expressed as pro-inflammatory markers, including serum amyloid A (SAA) (44). SAA subtypes 1–3 are well-described acute-phase reactants that are elevated in acute inflammatory conditions such as infection, tissue injury, and trauma. SAA subtypes have also been implicated in chronic metabolic diseases, including obesity, diabetes, and cardiovascular disease, and, passively, in autoimmune diseases, including systemic lupus erythematosus, rheumatoid arthritis, and inflammatory bowel disease (45). Circulating SAA levels are positively associated with visceral adiposity (46), suggesting that visceral fat is a potential source of SAA. These changes in circulating levels of adipokines are exactly SASP.

## 4 Obesity in cats

Obesity is the most common age-related disease in cats. Similar to that in humans, an increased incidence of obesity in cats has been observed to accompany aging in recent years (11, 47), and its prevalence is assumed to be 30–40% (47, 48). In cats, obesity is associated with the development of insulin resistance (49) and T2D (50, 51) and is considered a good model of human metabolic syndrome (52). Cats are more prone to obesity than dogs owing to their unique glucose and lipid metabolism characteristics (53, 54). In feline livers, glucokinase, the rate-limiting enzyme in glycolysis, is lacking (53), and gluconeogenic enzyme activity is higher than that in canine livers (55). Additionally, the expression levels of mRNA associated with the insulin signaling pathway, including insulin receptor substrate (IRS)-1, IRS-2, phosphatidylinositol 3-kinase (PI3K) P-85α, are significantly lower in cats than those in dogs (54), and expression levels of IRS-2 and PI3K mRNA significantly decreased in liver and skeletal muscle of obese cats (56). Furthermore, adiponectin, an adipokine that improves insulin sensitivity, is lower in cats in the normal state (54) and with weight gain (56). Thus, adiponectin appears to play an important role in the development of obesity-related metabolic disturbances in cats. Collectively, this evidence suggests that, similar to humans, cats have an inherently lower ability to process glucose and are predisposed to obesity and insulin resistance, as well as visceral obesity-induced lipid metabolism abnormalities.

Much like in human medicine, consensus on objective biochemical and mechanical parameters such as body mass index and its reference values for classifying weight status is lacking in veterinary medicine. Body condition score (BCS) is a commonly accepted semi-quantitative method for evaluating weight status. It involves subjective visual observation and palpation made by an observer, using a scale from 1 to 9, where 1 indicates emaciation, 5 is ideal, and 9 is extremely fat (57). Severely obese cats with a BCS of 9 showed excessive amounts of visceral fat accumulation and a significant increase in plasma concentrations of triglyceride, FFA, and TNF-α as pro-inflammatory markers and a significant decrease in adiponectin concentrations (10, 11). Aged obese cats with excessive visceral fat show enlarged adipocytes with macrophage infiltration (10, 11). mRNA expression levels of FAS and SREBP-1 in abdominal AT and livers of obese cats were significantly increased (58). In the aged obese cats, ectopic lipid accumulation was accelerated, and fatty liver is observed frequently (10, 11, 59). Changes in circulating levels of adipokines (adiponectin, leptin, and resistin), inflammatory cytokines (TNF-α, IL-1β, IL-6, MCP-1, and SAA) and lipids (FFA and triglyceride) in obese cats are summarized in Table 2 (59–62).

**Table 2:** Changes in circulating adipokines and inflammatory cytokines in obese cats.

Adipokines and inflammatory cytokines	References
Adiponectin ↓	(10, 11, 59, 60, 62, 87)
Leptin ↑	(60)
Resistin ↑	(60)
TNF-α ↑	(10, 11, 62)
IL-1β ↑	(62)
IL-6 ↑	(10, 11)
MCP-1 —	(61)
SAA ↑	(10, 59, 87)
FFA ↑	(11, 59)
Triglyceride ↑	(10, 11, 59, 87)

↑: increased, ↓: decreased, —: unchanged.

On the other hand, adipose tissue adiponectin mRNA and circulating adiponectin do not exhibit a correlation (63). Feline and human studies have shown that adiponectin gene expression is adipose depot-dependent (52), indicating that circulating adiponectin levels are dependent on other factors in addition to adiposity and fat depot location. Remodeling adipocytes are senescent cells and cannot produce adiponectin. This SASP is one of the characteristics of feline obesity and is considered to induce insulin resistance, followed by severe metabolic disorders such as obesity, diabetes, and vascular dysfunction, among others.

## 5 Intervention strategies for age-related obesity in cats

A healthy lifestyle has long been recognized as the most effective way to maintain health and prevent the deleterious effects of aging (64, 65). Adequate nutrition, moderate exercise, and a good mental state can effectively delay aging (66, 67). Balanced and adequate nutritional intake positively affects aging. Exercise is an efficient strategy for delaying aging due to various mechanisms, including DNA damage (68) and OS (69). Calorie restriction in animals is associated with a substantial reduction in pro-inflammatory markers in blood (70). Weight loss combined with exercise improves functional status, reduces some features of frailty in obese individuals, improves the cardiovascular risk profile, and reduces the risk of some types of cancer (71, 72). As aging progresses, living organisms experience a series of progressive degenerative changes and become more sensitive to internal and external stimuli, leading to OS aggravation, increased inflammation, apoptosis, and structural and functional cell and organ damage, resulting in a SASP followed by age-related diseases (73, 74). The SASP has been proposed as the underlying cause of inflammation and consists of various soluble factors, such as pro-inflammatory mediators (e.g., IL-6 and IL-8) and matrix-degrading molecules characterized by the release of pro-inflammatory cytokines (23). Alleviation of inflammaging will help prevent age-related diseases. The transcription factor NF-κB represents a promising target for SASP control. Incidentally, several NF-κB-dependent pro-inflammatory SASP factors are downregulated. Moreover, NF-κB is a key upstream regulator of the SASP and is, simultaneously, a transcriptional target of NF-κB (75). Metformin, an antidiabetic drug with pleiotropic effects, also targets senescent cells (76), negatively affecting NF-κB without affecting other inflammatory pathways such as p38 and JNK. Metformin-mediated inhibition of the SASP may contribute to the anti-aging effects observed after metformin treatment (77). Metformin activates AMPK and activated AMPK phosphorylates the acetyl-CoA carboxylase, inhibiting fat synthesis and promoting fat oxidation instead, thus reducing hepatic lipid stores and enhancing hepatic insulin sensitivity (78).

Various phytochemicals have been developed as senolytic drugs (12). Resveratrol, a natural polyphenol found in plants such as peanuts, grapes, and strawberries (79), modulates the expression of pro- and anti-apoptotic factors, neutralizes free radical species, affects mitochondrial function, chelates redox-active transition-metal ions, and prevents protein aggregation (80). Resveratrol inhibits the SASP through the SIRT1/NF-κB signaling pathway and delayed aging (81, 82) (Supplementary Figure 1). Quercetin (12) and curcumin (83) have shown anti-SASP and anti-inflammatory activities similar to those of resveratrol. For alleviation of inflammaging in age-related obesity cats, resveratrol supplementation (59), and quercetin supplementation (84) were effective. Metformin, which enhances peripheral insulin sensitivity and reduces hepatic glucose output, is used as anti-diabetic drug, however studies on metformin in age-related obesity cats are currently in progress (85).

Early diagnosis of the SASP by detecting various pro-inflammatory cytokines and inflammatory markers is possible (45, 86). In age-related obesity cats, SAA can be good diagnostic marker at early stage of inflammaging (87). Aging is inevitable in animals; however, delaying the onset of age-related diseases through adequate interventions in the early stage of the SASP is possible. Adequate nutrition, moderate exercise, and a good mental state can effectively prevent age-related diseases, including obesity, in cats.

## 6 Conclusion

Aging is characterized by chronic systemic inflammation, which is accompanied by cellular senescence, immunosenescence, organ dysfunction, and age-related diseases such as obesity, diabetes, vascular diseases, and even certain types of cancer. Senescent cells partially drive age-related tissue dysfunction by inducing a chronic SASP associated with various diseases. Obesity results in significant changes in the adipokine profile, creating a shift toward elevated levels of pro-inflammatory adipokines, such as leptin and resistin, and reduced levels of anti-inflammatory adipokines, such as adiponectin. Cats are more prone to obesity than dogs owing to their unique glucose and lipid metabolism characteristics. Severely obese cats show excessive visceral fat accumulation, a significant increase in plasma triglyceride, FFA, and TNF-α concentrations as pro-inflammatory markers, and a significant decrease in adiponectin concentrations. A healthy lifestyle is recognized as the most effective way to maintain health and fight the effects of aging. Adequate nutrition, moderate exercise, and a good mental state can effectively prevent age-related obesity in cats.

## Supplementary material

SUPPLEMENTARY FIGURE 1 Resveratrol activates AMPK in a SIRT1-dependent manner through deacetylation of LKB1. High dose of resveratrol activates AMPK directly and low dose of resveratrol activate AMPK via SIRT1, and stimulate mitochondrial biogenesis that results in improvement of lipid metabolism in tissues (81). LKB1, liver kinase B1; PGC-1α, peroxisome proliferator-activated receptor gamma coactivator1-alpha; NRF, nuclear respiratory factor; TFAM, transcription factor A, mitochondrial; NAD, nicotinamide adenine dinucleotide.
