# Supplementary figures and images for: Age-related obesity and inflammaging in cats
Source: Front Vet Sci. 2025 Oct 13;12:1639055. doi: 10.3389/fvets.2025.1639055 (PMC12554617; doi:10.3389/fvets.2025.1639055)

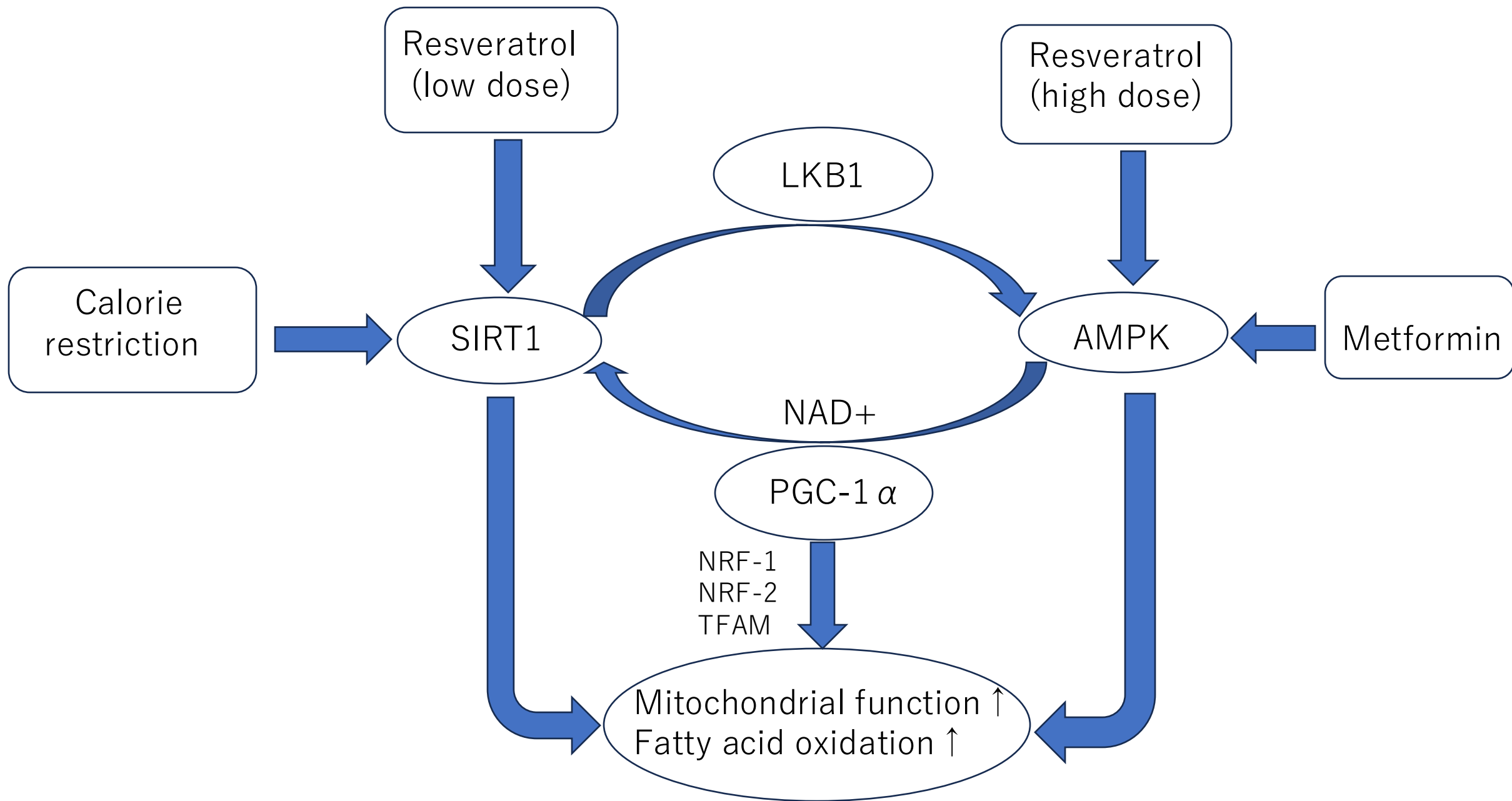

Supplement: SUPPLEMENTARY FIGURE 1 — Resveratrol activates AMPK in a SIRT1-dependent manner through deacetylation of LKB1. High dose of resveratrol activates AMPK directly and low dose of resveratrol activate AMPK via SIRT1, and stimulate mitochondrial biogenesis that results in improvement of lipid metabolism in tissues (81). LKB1, liver kinase B1; PGC-1α, peroxisome proliferator-activated receptor gamma coactivator1-alpha; NRF, nuclear respiratory factor; TFAM, transcription factor A, mitochondrial; NAD, nicotinamide adenine dinucleotide. [file Supplementary_Image_1.PDF]
